# T-cell depleted haploidentical hematopoietic cell transplantation for pediatric malignancy

**DOI:** 10.3389/fped.2022.987220

**Published:** 2022-10-14

**Authors:** Takuto Takahashi, Susan E. Prockop

**Affiliations:** ^1^ Pediatric Stem Cell Transplantation, Boston Children's Hospital/Dana-Farber Cancer Institute, Boston, MA, United States; ^2^ Department of Experimental and Clinical Pharmacology, University of Minnesota College of Pharmacy, Minneapolis, MN, United States

**Keywords:** immune reconsitition, cellular therapy, cliniMACS, stem cell transplant, children

## Abstract

Access to allogenic hematopoietic cell transplantation (HCT), a potentially curative treatment for chemotherapy-resistant hematologic malignancies, can be limited if no human leukocyte antigen (HLA) identical related or unrelated donor is available. Alternative donors include Cord Blood as well as HLA-mismatched unrelated or related donors. If the goal is to minimize the number of HLA disparities, partially matched unrelated donors are more likely to share 8 or 9 of 10 HLA alleles with the recipient. However, over the last decade, there has been success with haploidentical HCT performed using the stem cells from HLA half-matched related donors. As the majority of patients have at least one eligible and motivated haploidentical donor, recruitment of haploidentical related donors is frequently more rapid than of unrelated donors. This advantage in the accessibility has historically been offset by the increased risks of graft rejection, graft-versus-host disease and delayed immune reconstitution. Various *ex vivo* T-cell depletion (TCD) methods have been investigated to overcome the immunological barrier and facilitate immune reconstitution after a haploidentical HCT. This review summarizes historical and contemporary clinical trials of haploidentical TCD-HCT, mainly in pediatric malignancy, and describes the evolution of these approaches with a focus on serial improvements in the kinetics of immune reconstitution. Methods of TCD discussed include *in vivo* as well as *ex vivo* positive and negative selection. In addition, haploidentical TCD as a platform for post-HCT cellular therapies is discussed. The present review highlights that, as a result of the remarkable progress over half a century, haploidentical TCD-HCT can now be considered as a preferred alternative donor option for children with hematological malignancy in need of allogeneic HCT.

## Introduction

Allogeneic hematopoietic cell transplantation (HCT) is an established, potentially curative, option for some children with high-risk hematologic malignancy. HCT can mediate anti-tumor activity by virtue of cytotoxic agents in the conditioning regimen, eradication of stem cell populations with genetic predisposition to leukemia, and to different extents in different diseases, graft-versus-leukemia (GVL) activity. Historically the optimal allogeneic donor for HCT was considered to be an unaffected human leukocyte antigen (HLA)-matched related donor (MRD). However, an MRD is only available for 20%–30% of patients in need of HCT (1). Therefore, the use of alternative donors is essential to extend this curative option to the majority of patients in need. To support alternative donor transplantation, the first donor registry was established in 1974 and more than 32 million individuals have since been registered as potential HCT donors. Almost two decades later, the first umbilical cord blood bank was established in 1992. While transplant from matched unrelated donors (MUD) and umbilical cord blood (UCB) are established as options for patients lacking an MRD, they are associated with an increased risk of non-relapse mortality (NRM) largely due to an increase risk of graft vs. host disease (GvHD) and infection (2, 3). Similar to an HCT from an MRD, either bone marrow (BM) or peripheral blood stem cells (PBSC) can be collected from a MUD with potential accessibility of later repeated collection for adverse events (e.g., graft failure, relapse). However, the process is time-consuming involving identification, screening and collection of stem cells from an available and motivated donor, and may not meet the time constraints for recipients in urgent need of HCT. Moreover, HLA matched URDs are available for <30% in patients of racial/ethnic minorities in need as compared to 70%–80% of those of European descent (4). In UCB units are screened and stored frozen in banks and can be rapidly accessed. The biology of UCB allows a higher level of HLA mismatch such that recipients of racial/ethnic minorities are more likely to have an acceptable donor. While UCB unit expansion and double unit transplant are approaches to overcome the limited cell doses in UCB units, transplant with a single UCB is frequently not feasible in adolescents or young adults, and conventional UCBs do not offer the possibility of repeated infusions. Thus, there are a variety of issues that have limited the success of alternative donor transplantation, some of which can be overcome with the use of haploidentical related donors.

HCT from related individuals with a half-matched HLA profile (i.e., haploidentical HCT) can overcome the limitations of availability.

The major HLA genes are located on chromosome 6 and typically inherited without cross-over events. Thus, a patient will typically be haploidentical with each of their parents and half of their biological siblings. As a result, almost every patient has a readily available haploidentical motivated donor who can provide BM or PBSC repeatedly as necessary. However, haploidentical transplant requires specialized approaches to overcome the major immunological barrier of HLA mismatch with the potential of graft rejection mediated by recipient T cells and GvHD mediated by donor T cells. *Ex vivo* T-cell depletion (TCD) of grafts has been utilized in haploidentical HCT for decades to overcome this challenge (5). The first successful TCD transplantations were performed in infants with immune deficiency who were unable to reject HLA mis-matched stem cells. However, other early attempts of TCD haploidentical HCTs were complicated by graft failure. Even after engraftment was reliably achieved, the dependence on *de novo*, thymic derived T cell reconstitution has been associated with slow immune reconstitution. Recent advances in *ex vivo* TCD through sophisticated, targeted removal of specific lymphocyte subsets as well as *in vivo* TCD primarily with adoption of the post-transplant cyclophosphamide (PTCY) platform have led to improved outcomes. These approaches also eliminate or shorten the duration of immunosuppressive medications in the post-HCT period simultaneously offering a unique platform for adoptive cellular therapy for control of infectious complications and even underlying malignant disease in the post-HCT period. In this review, we discuss the evolution of TCD strategies in haploidentical HCT for pediatric malignancies with a focus on immune reconstitution and cell therapy post HCT.

## T cell depletion for haploidentical-HCT

### Evolution of *ex vivo* TCD

Recognized as a potentially fatal complication of haploidentical HCT since the 1970s and 1980s (6, 7), severe acute GvHD is a consequence of donor immune cell recognition of the recipient as foreign. As a strategy to prevent GvHD, T cell depletion was introduced over a half century ago and was initially successful in transplant of infants with impaired immunity. These early transplants were associated with a very limited incidence of acute and chronic GvHD, and TCD remain the most robust approach to limiting the incidence of chronic GvHD (5).

In this earliest version of *ex vivo* TCD, donor T cells were removed from bone marrow by sequential soybean lectin agglutination and E-rosette depletion (SBA-E-) resulting in a 3- to 4-log depletion of T cells (8). Subsequent approaches depended on the positive selection of CD34+ stem cells achieving a 5-log depletion of T cells successfully preventing GvHD and delivering high stem cell doses able to achieve reliable engraftment. However, even after issues of graft rejections were addressed, this approach was associated with an increased risk of treatment-related mortality (TRM) largely related to infection, such that prospective randomized trials failed to demonstrate improved OS (9).

Reconstitution of T cell immunity after transplant depends on homeostatic expansion of T cells infused with the graft and *de novo* thymic dependent T cell generation (10). Recipients of TCD HCT depend predominantly on the latter which takes a minimum of 3 months in young infants and up to several years in older adults (11, 12). A TCD HCT approach with a less complete depletion of T cells was an early attempt to solve this issue and was explored in a multi-center trial, but failed to demonstrate an advantage in disease-free survival (DFS) (13).

**Figure 1:**
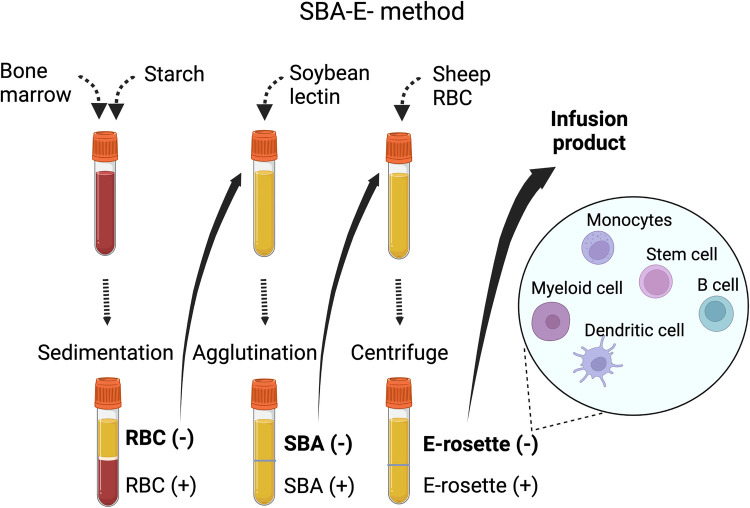
Soybean lectin agglutination and E-rosette (SBA-E-) method.There are four main steps in the procedure, (1) Filtered bone marrow is separated over hetastarch and the leukocyte-rich plasma (RBC-) is collected. (2) Leukocyte-rich plasma is mixed with soybean lectin, allowed to agglutinate and then settled through 5% albumin. (3) The unagglutinated (SBA-negative) population is mixed with sheep RBCs to allow R-rosette formation with T cells. After centrifugation over ficoll-hypaque, the supernatant (R-rosette negative) is collected. A final separation removes the trace of residual T cells in the SBA-E- fraction by separation of cells forming E-rosettes with neuraminidase-treated sheep RBCs.Abbreviation: SBA-E-, soybean agglutination negative and E-rosette negative; RBC, red blood cell.

**Figure 2:**
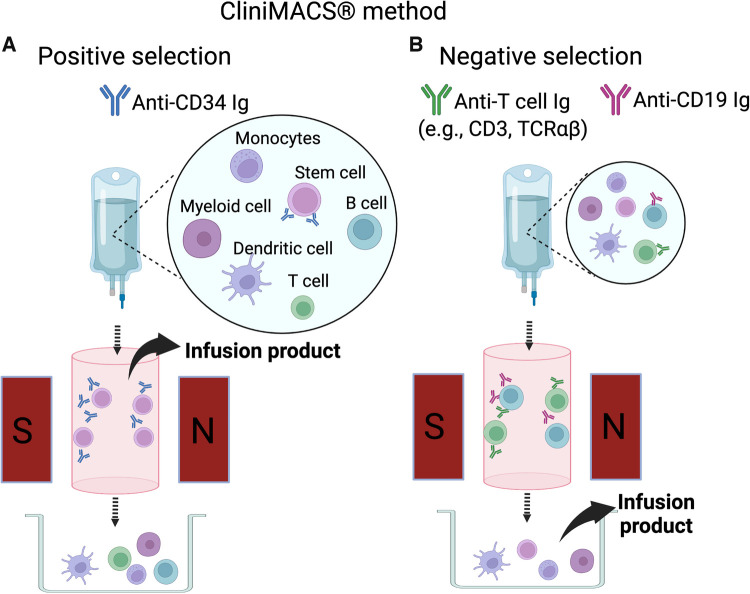
CliniMACS method. (**A**) The positive selection method (CD34+ selection). Donor marrow or G-CSF mobilized peripheral blood mononuclear cells are stained with anti-CD34 antibody conjugated with magnetic beads. CD34+ cells bound to the antibody-bead complex are captured in the magnetic field on the column. The cells retained in the magnetic field are released from the magnet and collected for infusion. (**B**) The negative selection method. Bone marrow or peripheral blood mononuclear cells are stained with anti-T cell antibody (e.g., anti-CD3 or anti-TCRαβ) and anti-CD19 antibody are used as antibody-magnetic bead complex. The cells bound to the antibody-bead complex are captured in the magnetic field. The cells not captured on the magnetic field of the column are collected for infusion.

Subsequently techniques were developed to deplete specific populations of T cells rather than positively select CD34+ stem cells. This approach can preserve the non-T cell composition of the graft and has allowed for increasingly sophisticated approaches to graft manipulation. Removal of CD3+/CD19+ cells successfully spared NK cells in addition to progenitor cells, which decreased the incidence of fatal infections (14). More recently, *ex vivo* depletion strategies selectively removing TCRαβ+ T cells or Naïve CD45RA+ T cells have been explored with promising results. These more sophisticated approaches to graft manipulation have the promise to improve post-transplant immune reconstitution while preserving the protection from GvHD; however, they are resource intense and will likely not be feasible in resource limited areas. In contrast, the use of PTCY for *in vivo* TCD of haploidentical HCT has emerged as an approach that is broadly accessible. Understanding the kinetics of immune reconstitution after haploidentical transplants facilitated with each of these approaches will be critical to successful implementation of the broadening armamentarium of alternative donor options. Key characteristics of different ex-vivo TCD methods are highlighted in Table 1, and clinical evidence is summarized in Supplementary Table S1. Figures 1, 2 show schematic illustration of SBA-E- and CliniMACS methods, respectively.

**Table 1:** Comparison of common *ex vivo* T-cell depletion methods.

Strategy	SBA-E-	CD34+	CD3-CD19-	TCRαβ-/CD19-	CD45RA+
Method	Differential sedimentation	CliniMACS	CliniMACS	CliniMACS	CliniMACS
Removed cells	T cells	T cells NK cells dendritic cells/ monocytes/myeloid cells	CD3+ CD19+	TCRαβ CD19+	CD45RA+
Retained cells	CD34+	CD34+	CD34+	CD34+	CD34+
	NK dendritic cells/ monocytes/myeloid cells		NK dendritic cells/ monocytes/myeloid cells	TCRγδ NK dendritic cells/ monocytes/myeloid cells	CD45RA- NK dendritic cells/ monocytes/myeloid cells
Graft source	BM or BM + PBSC	PBSC	PBSC	PBSC	PBSC
Stem cell dose in graft (cells/kg)	2–16 × 10^6^	8–15 × 10^6^	7–15 × 10^6^	9–15 × 10^6^	7–16 × 10^6^
T cell dose in graft (cells/kg)	CD3+: 0.01–0.6 × 10^6^	CD3+: 0.001–0.05 × 10^6^	CD3+: 0.01–0.06 × 10^6^	αβ+: 0.01–0.05 × 10^6^ γδ+: 8–11 × 10^6^	CD3+: 10–80 × 10^6^ CD3 + CD45RA+: 0.004–0.01 × 10^6^
Serotherapy	Yes	Yes	Yes or No	Yes	No
Post-HCT GvHD prophylaxis	None	None	Short course if higher residual T cells	None	Short course
Acute GvHD risk	+	+	++	+ (visceral or severe form are rare)	++
Chronic GvHD risk	+	+	++	+	++
Graft failure risk	+++ (+ for megadose)	+	++	+	+
Infection risk	+++	+++	++	+	+

Abbreviations: BM, bone marrow; GvHD, graft-versus host disease; HCT, hematopoietic cell transplantation; SBA-E-, soybean agglutinin negative E-rosette negative.

### T Cell Depletion by Soybean Lectin Agglutination with E-rosetting

Following the report by Dicke et al. in 1968 that splenocytes depleted of lymphoid cells by density centrifugation could engraft in irradiated mice, *ex vivo* TCD haploidentical HCT was introduced to the clinic in the 1980s (15). In 1981 Reisner and his colleagues at Memorial Sloan Kettering Cancer Center in New York, USA described a sequential technique to fractionate donor BM cells by initial removal of T cells by soybean lectin agglutinin, followed by elimination of residual T cells by E-rosetting (SBA-E-) with sheep red blood cells. This technique decreased donor T cells from the harvested BM graft by 1000-fold.

After durable engraftment of haploidentical TCD HCT was reported in two of three patients with primary immune deficiency, a large series confirmed this as a curative approach for infants with SCID (16, 17). However, this lectin-based TCD approach failed in patients with leukemia because of an unacceptably high incidence of graft rejection (as high as 50%) (18) mediated by the emergence of host-derived cytotoxic T cells with anti-donor specificity (19). These donor-specific host T cells can be overcome by donor T cells, but when uncontested, residual host T cells can eliminate the TCD graft. In HLA-matched sibling transplants, this barrier could be overcome by the addition of anti-thymocyte globulin (ATG) and/or thiotepa to the conditioning regimen (20).

Based on promising preclinical data (21, 22), the group at the University of Perugia, Italy used “megadose” haploidentical HCT to overcome host T cell-mediated graft rejection (23). After stem cell mobilization by G-CSF, both BM and PBSC were collected and depleted of T cells *via* lectin-based *ex vivo* TCD. These grafts contained 11.6 ± 4.7 × 10^6^ CD34+ cells/kg and were infused to patients after intensified conditioning with cyclophosphamide, thiotepa, ATG, and total body irradiation (TBI). Among the 17 adult and pediatric patients in this study, one had graft rejection and one had acute GvHD. However, fatal infections (3 patients with CMV and 2 with sepsis) and transplant-related toxicity (3 idiopathic pneumonitis and 1 GvHD) resulted in 9 deaths. This experience revealed the unacceptably high risk of severe infection in recipients of TCD HCT which was increasingly understood to be related to a prolonged period of immune compromise.

These lectin-based approaches have largely been replaced as they were technically challenging and thus difficult to export; however, they importantly demonstrated the feasibility of TCD HCT crossing HLA barriers and set the standard for subsequent approaches.

### Positive Selection of CD34+ Peripheral Blood Stem Cells

The advent of an immuno-magnetic cell separation device, CliniMACS (Meltenyi Biotec, Bergisch Gladbach, Germany) in 1999 was among the biggest breakthroughs in the field of haploidentical HCT. This device enables purification of desired stem cell subsets using microbeads bound to monoclonal antibodies recognizing specific cell surface markers. Using magnetic beads bound to anti-CD34 antibody, purity and recovery of collected CD34 cells were 98% and 75%, respectively with residual CD3 and CD19 cells of 0.03% and 1.2% (24). The University of Perugia group then investigated a protocol using CD34+ PBSC after GCSF mobilization in combination with de-escalation of conditioning regimen by replacing cyclophosphamide with fludarabine in 1999–2004 (25). Adults and children with hematologic malignancy received high-dose CD34+ cells (mean 13.8 × 10^6^ cells/kg) following conditioning with fludarabine, thiotepa, ATG, and TBI. No post-transplant GvHD prophylaxis was given. Of the 101 patients, 94 attained primary engraftment, and all but 1 engrafted after a second transplantation. Risks of acute GvHD, chronic GvHD, and NRM were relatively low (8%, 7%, and 37%, respectively). Despite a prior theoretical concern, the risk of relapse was not increased among those in remission prior to HCT (14%). A main setback of this regimen remained delayed immune reconstitution and a high risk of fatal opportunistic infection. In this cohort 71% (27/38) of NRM was attributed to infection, especially of viruses (14 cases of CMV, 1 EBV, 1 adenovirus, and 1 HHV-6). In a recent randomized trial in adult patients with AML or MDS undergoing HLA-matched HCT, it was demonstrated that delayed immune reconstitution associated with an increased incidence of fatal infections led to a trend of higher rate of NRM (9).

These results were extended to pediatric patients in a retrospective study from the European Blood and Marrow Transplantation reporting on 102 recipients of TCD haploidentical HCT transplanted between 1995 and 2004 (77% used CD34+ selection by CliniMACS) (26). Primary and secondary graft failure were observed in 9% and 4% of patients, respectively. Acute Grade 2, Grade 3–4, and chronic GvHD occurred in 13%, 9%, and 14% of patients, respectively. Five-year leukemia-free survival, relapse, and NRM were 27%, 36%, and 37%, respectively. A trend of improved leukemia-free survival and decreased relapse was observed in recipients of a graft with a higher number of CD34 cells (adjusted *p* = 0.09 and *p* = 0.07, respectively). This study demonstrated that relapse and infection are common causes of mortality (46% and 33% of death, respectively). Aversa et al. investigated nonmyeloablative haploidentical HCT with ATG/Fludarabine/TBI 2Gy regimen followed by infusion of megadose CD34+ cells by CD3-/CD19- TCD combined with PTCY as the sole posttransplant GvHD prophylaxis (27).

In 1999 Small et al. demonstrated the improved overall and event free survival in younger recipients of TCD HCT and evaluated the risk of infection as related to attaining milestones of immune reconstitution. The importance of achieving specific milestones of immune reconstitution was again demonstrated by Admiraal et al. in 2015 who established that achieving a CD4 of 50/μl by day 100 post HCT was associated with improved outcomes (28). This milestone was validated in 2020 by van Roessel and Prockop et al. who demonstrated that those undergoing TCD HCT with any of the above methods were less likely to achieve this milestone, but those who did achieve it similarly had superior OS (12). More recently, de Koning et al. reported that pediatric patients who developed GvHD regardless of the transplant platform had improved survival if they had attained this milestone (29).

### Selective Depletion of CD3+ T Cells and CD19+ B Cells

The appreciation for the essential roles played by non-T/non-B cells in the success of HCT was important in shifting from a positive selection of CD34+ HSCs to an approach in which CD3+ and CD19+ cells are removed from the graft by “negative selection”, sparing other populations including NK cells, dendritic cells, and monocytes. In particular, many preclinical studies suggested contribution of NK cells to successful engraftment, immune reconstitution, and anti-leukemia effect (30).

Diaz et al. reported experience with CD3-/CD19- haploidentical PBSC with fludarabine-based reduced intensity conditioning (RIC) among 70 children with hematologic malignancy in Spain between 2005 and 2013. Pharmacologic GvHD prophylaxis consisted of a short course of cyclosporine (tapered before Day +30) (14). Graft failure were observed in 4 patients (6%), 3 of whom were rescued by a second transplant. With a median follow-up of 22 months the probability of NRM, relapse, and disease-free survival (DFS) were 23±5%, 32±6%, and 52±6%, respectively. Recovery of NK cells were notably faster compared to recipients of CD34+ PBSC (31), and infection-related mortality was 9%. However, this regimen was associated with a higher incidence of acute and chronic GvHD (grade 2–4 acute 35±5% and chronic GvHD 46±7%). This insufficient GvHD prophylaxis was attributed to higher residual T cells as compared to CD34+ PBSC (31).

Lang et al. at University of Tübingen in Germany also investigated CD3-/CD19- haploidentical PBSC using a melphalan-based non-TBI RIC regimen in pediatric patients with hematologic malignancies treated between 2004 and 2012. Median CD34+ and CD3+ cells in the infused graft were 15 × 10^6^/kg and 0.06 × 10^6^/kg, and those with CD3+ cells >0.025 × 10^6^/kg received a short course of mycophenolate mofetil (MMF). This trial had a 12% incidence of graft failure, where all were rescued by a repeated transplantation, and 5-year TRM of 20% (causes of death included bacterial infection [*n* = 1], GvHD [*n* = 2], cardiomyopathy [*n* = 2]). However, the incidence of relapse was high even among those who underwent the first HCT in remission (2-year relapse incidence and 3-year EFS were 38% and 46%, respectively). Acute GvHD grade 2–4 (27%) and chronic GvHD (21%) resulted in two deaths.

### Selective depletion of TCRαβ+ T cells and CD19+ B cells

More recently, further refinements of negative selection *via* CliniMACS have been introduced. It has been recognized since the 1990s that different subsets of T cells play critical roles in immune regulation post HCT. Several lines of evidence suggested that TCRαβ+ populations are more central than TCRγδ+ populations in causing GvHD. Biopsies of skin, liver and intestines in the presence of clinical GvHD showed tissue infiltrating lymphocytes that were almost exclusively TCRαβ+ with very few TCRγδ (32). Moreover, the number of TCRγδ cells in tissue affected by GvHD are comparable to those of controls without GvHD (33). Another study showed a correlation between higher TCRαβ cell dose in the graft and the risk of acute GvHD, while a higher dose of TCRγδ was correlated with successful engraftment (34). These findings have been confirmed in a murine model (35).

In 2007 Chaleff et al. from St. Jude Children's Research Hospital developed a technique to perform clinical-scale TCRαβ/CD19 depletion using the CliniMACS device (36). They successfully reduced the total T cell dose by 4–5 log, which is comparable to CD34+ TCD (37). The group in Rome, Italy was among the first to employ this technique depleting TCRαβ/CD19 from haploidentical PBSC in the treatment of 13 children with nonmalignant diseases (38). Lang, et al. from Tubingen, Germany subsequently reported their experience in a pediatric cohort transplanted for both malignant and non-malignant disease (39). These early single-center reports revealed a high rate of engraftment, a very low risk of visceral acute or chronic GvHD, and early reconstitution of TCR γδ+T cells and NK cells (39, 40).

These favorable results of TCRαβ/CD19 depleted HCT were confirmed in multicenter experiences. In an Italian registry of 13 centers, HCT outcomes were compared in children with malignancy who underwent myeloablative haploidentical HCT with TCRαβ-/CD19- and conventional T-cell replete HCT from matched or mismatched URDs (*n* = 98, 127, and 118, respectively) (41). In this first multicenter study of TCRαβ-/CD19-, all approaches showed a very low incidence of graft failure (2% each). Depletion of αβ T-cells in combination with ATG without other pharmacological GvHD prophylaxis eliminated any case of grade 3–4 or visceral acute GvHD. Incidences of grade 2 acute GvHD and extensive chronic GvHD were also substantially decreased (16% and 1% in TCRαβ-/CD19- vs. 35% and 3% in matched URD, and 44% and 12% in mismatched URD). In addition, combined *ex vivo* and *in vivo* B cell depletion with CD19- selection and a single dose of rituximab completely prevented post-transplant lymphoproliferative disease. Third, TCRαβ-/CD19- haploidentical HCT, in comparison to matched URD, was associated with higher CD34+ cell doses (14.4 vs. 5.2 × 10^6^ cells/kg), faster neutrophil recovery (13 vs. 19 days), and faster platelet recovery (11 vs. 22 days). Overall, NRM was comparable in haploidentical TCRαβ-/CD19- HCT and matched URD HCT (6% vs. 9%). However, disease relapse remained the primary cause of failure in this relatively low-risk cohort (i.e., pediatric leukemia in remission with myeloablative regimen). Incidence of relapse was comparable between recipients of TCRαβ-/CD19- HCT and recipients of matched URD HCT (26% vs. 29%) despite a lower proportion of first complete remission in the former.

### Selective depletion of naïve (CD45RA+) T cells

Another approach to deplete targeted populations of T cells is the removal of CD45RA+ cells, which selectively depletes naive T cells while retaining memory T cells. Naive T cells are known to possess higher alloreactive potential and contribute to GvHD development (42), while the latter augments diversity of T cell population against virus infection. On these bases, Bleakley et al. from Fred Hutchinson Cancer Center (Seattle, USA) developed a 2-step graft manipulation technique that incorporates a CD34+ positive component with one depleted of CD45RA+ cells (43). It is hypothesized that addback of T cells selectively depleted of CD45RA+ cells will improve immune reconstitution and reduce severe viral infection without significantly increasing the risk of GvHD. In their phase 2 single-arm trials including 138 adults with hematology malignancy undergoing MRD or MUD HCT, this method resulted in limited acute GvHD (4% and 0% for grade 3 and 4, respectively) albeit with a high incidence of acute GvHD grade 2 (71%) and remarkably infrequent chronic GvHD (total 7%, moderate 1%, severe 0%) (44). Memory T cells in the graft resulted in rapid T cell recovery and transfer of protective virus-specific immunity. Excessive rates of infection or relapse did not occur and OS was 78% at 2 years.

Triplett et al. at St. Jude Children's Research Hospital reported the efficacy and safety of CD45RA- HCT in pediatric malignancy as compared to CD3- HCT. Their regimen incorporates a CD34+ TCD graft on Day 0, followed by a CD45RA depleted stem cell infusion on Day +1, and a subsequent NK cell infusion on Day +6 (45). The CD45RA- method, in comparison to the CD3-, attained 2000-fold higher donor T cell doses and higher T-cell counts at Day +30 post-HCT (550/μl vs. 10/μl; *p* < 0.001), and higher T-cell diversity by Vβ spectra typing at Day +100 (*p* < 0.001). The CD45RA- method also resulted in a significant reduction in both the incidence and duration of any viremia (CMV, EBV, or adenovirus) in the first 6 months post-HCT. Although incidence of grade 3–4 acute GvHD were comparable in both types of TCD-HCT (23.1% vs. 22.6%), this risk was higher than that reported with other methods of TCD (e.g., CD3-CD19- or TCRαβ-/CD19-). Of note, this protocol used a short course of MMF (day <60) and total lymphoid irradiation to replace serotherapy and decrease the risk of graft rejection.

While it is clear that prevention and management of relapse is still a relevant and challenging problem that limits the success of HCT, the sophisticated graft engineering employed in haploidentical HCT can provide a unique platform for post-transplant therapies.

### *In vivo* T cell depletion

Two approaches to support haploidentical transplant with *in vivo* TCD have been developed. Neither of these depend on sophisticated graft engineering and thus are easily exportable globally including to resource restricted regions. The first PTCY was pioneered by the group at Johns Hopkins Hospital (Baltimore, MD, United States), and depends on the preferential sensitivity of activated T cells to cyclophosphamide. This platform initially used a reduced intensity conditioning regimen combined with a conventional marrow graft and post-transplant (days 3 and 4) high dose cyclophosphamide combined with a CNI and MMF for GvHD prophylaxis (46) with reports of similar incidence of GvHD to that seen following a conventional MUD transplant in adults for AML (47). Subsequently myeloablative conditioning (48, 49) and the use of peripheral blood mononuclear cells (48) have all been explored as recently reviewed (50). A recent retrospective report from the EBMT on pediatric patients transplanted with PTCY based regimens for ALL reported a 2-year cumulative incidence of relapse of 25%, 37% and 50% for those transplanted in 1st, 2nd, or ≥3rd CR respectively and a 2-year OS, leukemia-free survival and GvHD-free, relapse-free survival (GRFS) of 50.8%, 38.5% and 29.2% (51).

The second approach to *in vivo* TCD has been termed the Beijing Protocol. For malignant disease both TBI-based on non-TBI based myeloablative regimens with high doses of ATG and GvHD prophylaxis with MMF, CSA and MTX are used with infusion of a G-CSF primed bone marrow graft. Most recently, preliminary reports suggest that this approach can be combined with a PTCY regimen. Promising results from pediatric and young adult cohorts of patients with Ph+ and Ph- ALL including those with MRD at the time of transplant (52, 53). However, beyond publications referencing a high incidence of CMV reactivation and refractory CMV infection (54), there is scant information related to immune reconstitution.

PTCY and Beijing protocols for *in vivo* TCD require post-transplant calcineurin inhibitor based immune suppression and are associated with distinct toxicities and sequence of immune reconstitution. The requirement for post-transplant immune suppression makes them less ideal as platforms for adoptive cellular therapy in recipients of haploidentical HCT and as such they are discussed only briefly.

## Engraftment and immune reconstitution after haplo-HCT

### Overall

Delayed immune reconstitution has been recognized as a major impediment to the success of T cell depleted transplantation. While an improved understanding of the contribution by different populations of the immune system to protective immunity has led to more sophisticated approaches to graft manipulation, the ability to ensure that protective immunity is reestablished in the post-transplant period remains elusive. Immune reconstitution is a complex process involving factors in the recipient (e.g., age, underlying disease), the donor (e.g., graft source cell dose, HLA match, sex match, CMV match, age) HCT regimen (e.g., conditioning regimen, *in vivo* T cell depletion, post-HCT GvHD prophylaxis), and post-HCT complications (e.g., CMV reactivation, GvHD) (55). The pattern of immune reconstitution after conventional MRD HCT proceeds from monocytes to neutrophils and NK cells within 1 month, with reconstitution of T cells starting at around 2–3 months, and B cells being the slowest to recover at around 1-year post HCT. In contrast after TCD HCT, T cell reconstitution, specifically CD4 T cell reconstitution is typically the last component of immunity to recover.

### Monocytes

Monocytes are the first cell population that recover after an HCT. Although the kinetics and clinical implications of monocyte recovery are not as well-known as other populations, some studies suggest favorable HCT outcomes in patients who have early monocyte recovery. In a heterogenous HCT cohort with various donors (MRD, MUD, and single or double UCB) and various conditioning regimens (MAC or RIC) early recovery of monocytes at 1-month post HCT was associated with better OS, relapse, and NRM (56, 57). In TCD haploidentical HCT donor monocytes and granulocytes are depleted by CD34 positive selection but not by CD3/CD19 negative selection (<1% vs. 130 × 10^6^ cells/kg) (58). However, another comparative study revealed similar monocyte reconstitution by TCD technique (CD34+ vs. CD3-/CD19-) or conditioning intensity (MAC vs. RIC). In this cohort of pediatric hematology malignancy, monocyte reconstitution was swift; approximately 80% achieved their age norm value at 30 days after SCT (59).

### Neutrophil

Neutrophil recovery has been among the most established early indicators of HCT success which is also associated with risk of serious infection. In early experience with “megadose” TCD myeloablative haploidentical HCT by SBA-E- technique, median time to neutrophil recovery >1,000 was 11 days (range 8–19) by 10.6 × 10^6^ ± 5.4 CD34+ cells/kg with G-CSF use (8). In a study with CD34+ positive selection method (majority by CliniMACS) for myeloablative haploidentical HCT in pediatric malignancy, median time to neutrophil >500 cells/μl was 15 days (range, 8–55) without G-CSF, with a CD34+ cell dose of 12.3 (range, 1.4–95) × 10^6^ cells/kg (26). Similarly, with a median 7.0–9.2 × 10^6^ CD34+ cells/kg neutrophil recovery was 13 days (range, 7–20) in the CD3-/CD19- haploidentical HCT with RIC for pediatric malignancy (14, 60). Likewise, median time to neutrophil >500 cells/μl was 13 days (range, 6–23) without G-CSF in TCRαβ-/CD19-, which was significantly faster than MUD or MMUD by T-replete methods (median 19–20 days) in myeloablative HCT in pediatric malignancy. Notably, the infused CD34+ cell dose was much higher in TCRαβ-/CD19- than in MUD or MMUD (median 14.4 vs. 5.2 vs. 4.9 × 10^6^ cells/kg) (41). In another early-phase clinical trial median neutrophil recovery was also swift (median 10 days, range 9–13) in CD45RA- TCD method which contained median 6.4 × 10^6^ CD34+ cells/kg (45). Together, it has been reported consistently that TCD haploidentical HCT, which commonly utilized G-CSF-mobilized PBSC, has early neutrophil recovery comparable to matched donor PBSC. PTCY with RIC conditioning using BM graft was shown to attain neutrophil recovery was 15 days (range 11–42) (46).

### NK cells

NK cells are another component of innate immunity that contributes to immune reconstitution in the first month after HCT. NK cells play critical anti-tumor and anti-infection roles in the haploidentical TCD HCT where potent donor T cell-mediated effects are absent. NK cells are potentially preferable to donor T cells because of lower alloreactivity, albeit with conflicting data (30). NK cells develop self-tolerance (i.e., “licensed NK cells”) when their inhibitory surface receptor [i.e., killer-cell Ig-like receptor (KIR)] encounter matching HLA-class I ligand. These licensed NK cells can elicit potent activity against leukemia cells that do not express matching ligand (i.e., KIR receptor-ligand mismatch) (61–63). In pediatric leukemia patients undergoing haploidentical HCT NK cell reconstitution was faster in CD3-/CD19- as compared to CD34+ selection (350 vs. 180 cells/μl, respectively, on Day +60) (31), which reflects depletion of NK cells from the graft in the latter but not in the former. Similarly, TCRαβ-/CD19- method also retains NK cells in the graft and attains comparably fast reconstitution (approximately 250 cells/μl at Day 30) (64, 65). NK cell recovery is delayed in haploidentical HCT using PTCY because of elimination of donor NK cells by PTCY administration after initial proliferation. Normal NK phenotype is recovered at 9 to 12 months post HCT (66). This contributes to suppressed GVL by donor NK cells in PTCY setting.

### T cells

Reconstitution of protective donor T cell function without alloreactivity is a hallmark of a successful allogeneic HCT. Higher CD3+, CD4+, or CD8+ T cells at Day +100 are associated with favorable NRM and OS (12, 67). Recently a milestone of 50 CD4 T cells/μl was defined as a predictor of improved OS, NRM and DFS in pediatric and young adult recipients of conventional TCD, and cord blood transplant (12, 28). In this study, recipients of TCD HCT less frequently attained this target CD4+ count.

Salzmann-Manrique et al. developed a mathematical model and predicted reconstitution of CD3, CD4, CD8 cells in myeloablative HCT with combined *ex vivo* and *in vivo* TCD. The predicted T cell counts at Day +90 by CD34+ vs. CD3-/CD19- methods were 154 vs. 135 cells/μl for CD3+, 31 vs. 44 cells/μl for CD4+, and 67 vs. 89 cells/μl for CD8+, respectively (59). These T cell counts correspond to 3%–16% of the counts in the age-matched healthy controls. Their infused CD3+ cell doses were 1.0 vs. 8.9 × 10^3^ cells/kg respectively. Better T cell reconstitution was reported by Bertaina et al. with TCRαβ-/CD19- TCD combined with *in vivo* TCD in 98 children with pediatric malignancy (41). In comparison to CD34+ or CD3-/CD19- technique, this method results in a 3–4 log higher infused dose of γδ+ T cell with minimal αβ+ T cells (8.1 and 0.04 × 10^6^ cells/kg respectively). At 3 months post TCRαβ-/CD19- HCT, mean CD3+ count was approximately 400 cells/μl, CD4+ was 100 cells/μl, and CD8+ was 250 cells/μl. In comparison to T cell replete URD HCT, CD3+/CD4+/CD8+ cell counts were all lower 30 days after TCRαβ-/CD19- HCT, but comparable counts were achieved by Day +180 for CD4+, and Day +365 for CD3+ and CD8+ cells. Moreover, detailed immunology analysis in TCRαβ-/CD19- by Airoldi et al. revealed that peripherally expanded infused γδ T cells are the predominant T-cell population in patients during the first weeks after HCT (91.5% of CD3+ cells) (68). Triplett et al. reported even faster T cell reconstitution and higher CD3+ cell dose in their experience with CD45RA- HCT (69). Their TCD protocol is also among rare TCD methods that do not include *in vivo* TCD potentially eliminating the risk of prolonged exposure to serotherapy impairing graft derived immune reconstitution. With the infusion of a median of 122 × 10^6^ CD3+ cells/kg mean CD4+ count was approximately 150 cells/μl by Day +30 and 200 cells/μl by Day +90 in these patients. Collectively these studies illustrate that selectively depleting only subsets of T cells (i.e., TCDαβ+ or CD45RA+ cells), can translate into more rapid immune reconstitution. In haploidentical HCT with PTCY, proliferating donor T cells are depleted by infusion of PTCY, except for regulatory T cells, which are resistant to cyclophosphamide because of higher expression of detoxifying enzyme, aldehyde dehydrogenase. Without mature donor T cells, naive T cell-driven immune reconstitution is slow and T cell counts may be undetectable up to 6 weeks post HCT (70), the period with MMF in the typical PTCY regimen. In contrast regulatory T cells continue to expand and achieve the normal donor level at day 30 (71).

### B cells

The B cell population is the slowest to reconstitute after HCT, and may take a few years to reach the normal levels. In Salzmann-Manrique's mathematical model CD19+ B cells in CD34+ selected pediatric myeloablative HCT are predicted to start recovering at Day +60 (45 cells/μl) and subsequently plateau from Day +90 through Day +365 (92–105 cells/μl). In contrast, B cell recovery was slower in recipients of HCT with the CD3-/CD19- method; CD19+ B cells stayed at 20–40 cells/μl from Day +60 to +365. However, in a multicenter study with TCD by TCRαβ-/CD19-, despite use of rituximab on Day -1, Bertaina et al. reported a faster recovery to approximately 80 cells/μl at Day +90 and, by Day +365, reached a level that is comparable to MUD and MMUD (approximately 400 cell/μl) (41). PTCY approaches tend to have relatively fast B cell recovery by day 49 to 77 post HCT (71, 72); however, most of these B cells are naive up to day 180 (72).

## Haploidentical HCT as a platform for adoptive cell therapy

### Overall

As regimens for *ex vivo* TCD-based haploidentical HCT require minimal post-transplant immune suppression (26, 64), this approach can provide an ideal platform for posttransplant cell therapy. As one of the major complications of *ex vivo* TCD HCT is delayed immune reconstitution, an appealing solution is the adoptive transfer of antigen specific or otherwise tolerized populations of T cells. Donors of haploidentical HCT (i.e., family members) are readily available for repeated apheresis for either planned or need-based post-HCT cellular therapies. Thus, various cellular therapy approaches have been explored in the setting of TCD haploidentical HCT.

### Unmanipulated T cell addback

Addback of donor T cells after HCT has been investigated as a means to overcome delayed immune reconstitution and resultant high risk of fatal infection. When added to a CD34+ TCD haploidentical HCT, a Phase 1 study in adults using prophylactic DLI at a dose of 3 × 10^4^ T cells/kg resulted in an unacceptably high rate of acute GvHD (73), but more promising in a pilot study, Oved and colleagues demonstrated that targeted add-back to achieve a dose of 1 × 10^5^ T cells/kg with a CD3-/CD19- TCD graft was feasible and associated with acceptable incidence of GvHD (74). Alternatively, delaying infusion of T cell add-back has been explored (73, 75). Gilman et al. reported on a phase 1/2 study of 5 × 10^4^ haploidentical T-cells/kg administered 30–42 days after transplant and followed by methotrexate with or without rituximab in pediatric recipients of CD34+ TCD haploidentical HCT. The study met the primary objective with 67% of patients having CD4+ cells >100/μl by Day +120 with acceptable rates of GvHD (Grade 2–3 11%, no Grade 4 GvHD, and 16% chronic). Central to this approach is the role that acute tissue injury induced by conditioning plays in the induction of GvHD (76). Delaying the adoptive transfer of allogeneic T cells can decrease the inflammatory stimulus that these T cells encounter. Additionally, this approach avoids exposing the adoptively transferred T cells to residual effects of serotherapy (e.g., ATG or alemtuzumab) that are commonly utilized in TCD-HCT (77, 78).

### DLI with safety switch

In order to minimize the risk of GvHD associated with infusion of HLA disparate T cells, genetic engineering of donor T cells with suicide genes enables a selective *in vivo* depletion of donor T cells if GvHD develops.

T cells transduced with the herpes simplex virus thymidine kinase (HSVtk) can be eliminated in the event of GvHD by administration of ganciclovir, which is intracellularly converted by HSVtk to a toxic metabolite and induces arrest of DNA synthesis and subsequent cell death. A limitation of this method is the cell-cycle specificity; this ganciclovir-induced HSVtk-T cell depletion is restricted to the cells in proliferation cycle, which may allow prolonged survival of cells in the other cell-cycles. Nonetheless, the safety and activity of this method were shown in a phase 1/2 study for adults who underwent CD34+ TCD haploidentical HCT after myeloablative conditioning with ATG. No post-HCT GvHD was given. Patients received monthly doses of 10^6^ -10^7^/kg of HSVtk T cells starting Day +28. Twenty-two of the 28 patients (79%) attained T cell count >100 cells/μl at median of 23 days (range 13–42) after HSVtk T cell infusion. These T cells included antigen-specific T cells against CMV and EBV. Administration of gancyclovir successfully controlled all patients who developed acute (*n* = 10) or chronic (*n* = 1) GvHD.

More recently, the inducible caspase 9 (iCasp9) system has been explored. The iCasp9 transgene consists of a drug-binding domain and a linker to human caspase 9. Caspase 9 is activated by administration of AP1903, a small molecule that specifically binds to this receptor, and subsequently induces apoptosis of the transduced cells. Unlike HSVtk, activation of the iCasp9 safety switch is cell-cycle independent and can eliminate the target cells rapidly. Haploidentical HCT with T cells transduced with iCasp9 has been investigated by Brenner et al. at Baylor College of Medicine (Houston TX USA). In their initial clinical experience, 5 children with relapsed leukemia underwent haploidentical HCT with iCasp9-transduced T cell infusion. A single dose of AP1903, given to four patients with active GvHD, eliminated >90% of the modified T cells within 30 min after administration and resolved their GvHD without recurrence. The group later reported the sustainability of iCasp9-engineered T cells in a larger cohort of pediatric haploidentical HCT recipients (79). iCasp9 T cells continued to expand after the infusion, peaking at 9 months, and persisted for at least 2 years (80).

### Elimination of alloreactive donor T cells

Another strategy to augment the T cell repertoire after CD34+ TCD haploidentical HCT without an unacceptably high risk of GvHD is *ex vivo* depletion of alloreactive T cells. After co-culture of donor T cells with irradiated recipient PBMCs two approaches to depletion have been explored. The first uses anti-CD25 antibody-toxin conjugate (81, 82) to remove CD25+ activated T cells. This technique successfully reduced the proportion of alloreactive T cells to <1% with a low risk of Grade 2–4 acute GvHD (0%–13%), and early reconstitution of CMV and EBV specific immunity was achieved. The second approach pioneered by Roy et al. depends on selective uptake by activated T cells of the photosensitizer TH9402 (83, 84). In their phase 1 single-center and phase 2 multicenter dose-escalation studies in adults, photo-depleted donor T cells were infused at a median of 28 days post HCT. None of the patients developed grade 3–4 acute GvHD and TRM at 1 year was lower than that in a historical cohort of recipients of CD34+ haploidentical TCD (32.2 vs. 70.3%) (83). A phase 3 randomized control trial is currently underway comparing haploidentical HCT with photodepleted T cells to T-cell replete PTCY transplant platform (NCT02999854).

Induction of anergy by blocking B7/CD28 signaling in potentially alloreactive donor T cells is another approach to minimizing the risk of GvHD. Blockade of B7 interactions by either anti-B7-antibody or a CTLA4-Ig fusion protein can induce T cell anergy in an *ex vivo* culture. A phase 1 dose-escalation study with administration Day +35 - Day +42 after haploidentical HCT demonstrated earlier (2–3 months vs. 9 months) reconstitution of functional virus-specific immunity at higher (10^3^–10^4^/kg) vs. lower (10^2^/kg) dose levels. However, Grade 2–4 GvHD developed in 5 of 12 patients at the high doses compared to 0 of 4 at the lower dose (85). Another trial used CTLA4-Ig-induced anergy in 12 children and young adults. All 5 surviving patients attained CD4 count >400/μl by 6 months but GI acute GvHD developed in 3 patients (86). Subsequently, these authors identified expansion of regulatory T cells as potentially contributing to their results (87). Most recently, CTLA-4-Ig blockade has been approved as prophylaxis in the setting of conventional URD HCT. Collectively, anergy induction remains a promising strategy for GvHD prophylaxis.

### Antigen-specific donor T cells

Infusion of selected subsets of T cells has been explored to provide more tailored T cell addback. Prophylactic or pre-emptive infusion of *ex vivo* engineered antigen-specific donor T cells can enhance immunity against specific infectious pathogens or leukemia cells (88–93). However, donor-recipient HLA mismatch can limit the efficacy of this approach due to the constraints of immunodominance. When antigen-specific donor T cells are restricted by HLA alleles not shared by the recipient, the donor T cells will not recognize recipient cells infected with the target virus or leukemia cells derived from the recipient. That said, haploidentical donor derived virus-specific T cells have been shown to be effective against various pathogens (e.g., CMV, EBV, adenovirus, Aspergillus) with a minimal risk of GvHD (89, 90, 93). In contrast clinical use of leukemia-specific T cells recognizing tumor associated antigens has not been as successful, likely because these cells are low frequency and predominantly naive making *ex vivo* expansion difficult. Nonetheless, approaches for generating tumor specific T cells from HLA identical and non-identical donors are being developed. After HLA-matched HCT, promising activity was shown for WT-1 specific donor T cells (Wilms tumor antigen 1). In preclinical studies Wiebking et al. developed a system for generating haploidentical donor derived, TCR knock-out, CD19-specific chimeric antigen T cells (94).

## Optimizing regimens

Delayed CD4 cell reconstitution with higher ATG exposure post HCT infusion has been investigated by Boelens and Admiraal et al. in various types of HCT in children using ATG (28). Subsequently, individualized ATG dosing regimen informed by the previous study was tested prospectively in pediatric URD HCT and showed improved early immune reconstitution (95). Similar exposure-response correlation was reported in CD34+ TCD by the same group (96). The risk for delayed CD4 reconstitution was shown with higher ATG exposure post HCT [>100 arbitrary unit per day/milliliter (AU·day/ml)] in BM and PBSC (28), while the thresholds were much lower in UCB and CD34+ TCD (>20 and >30 AU·day/ml) (28, 96). Conversely higher ATG exposure resulted in lower risk of acute and chronic GvHD (28, 97). Another group in Japan reported a similar positive correlation between day 0 ATG exposure and risk of acute GvHD in a myeloablative haploidentical regimen (98).

## Future directions and conclusion

Despite the remarkable improvement in outcomes of TCD haploidentical HCT, further research is needed to provide more potent anti-leukemia immunotherapy for very high-risk malignancy that has an ominous prognosis with conventional therapeutic options. As reviewed, novel approaches that can capitalize on the haploidentical platform, especially given that many recipients do not require pharmacologic GvHD prophylaxis in the post grafting period, are being developed. In contrast regimens with less therapy-related toxicity would be desired in patients with lower-risk of relapse. In addition TCD haploidentical HCT can potentially be offered to very different group of patients, solid organ transplant recipients. Minimal GvHD risk without posttransplant prophylaxis can make TCRαβ- TCD an ideal regimen when combined HCT/solid organ transplant from the same haploidentical donor is pursued for induction of long-term tolerance in the transplanted solid organ (99).

In summary, an iterative progress in tailored graft engineering has made TCD haploidentical HCT a more optimized therapeutic modality. Current trials are exploring approaches to improve post-HCT immune reconstitution.

With its inherent advantage in donor accessibility, this achievement has resulted in a paradigm shift in donor selection for pediatric malignancy, where haploidentical donors can now be considered as a favorable option for the majority of patients in need of HCT. In particular, the low risk of severe chronic GvHD in TCD HCT is important in children to prevent life-long consequences. TCD haploidentical HCT also offers an ideal platform for various post-HCT adoptive cellular therapy to either overcome and/or augment its effects.
