# Supplementary material for: T-cell depleted haploidentical hematopoietic cell transplantation for pediatric malignancy
Source: Front Pediatr. 2022 Oct 14;10:987220. doi: 10.3389/fped.2022.987220 (PMC9614427; doi:10.3389/fped.2022.987220)
Supplement: Supplementary file 1 [file Table1.docx]

**Supplemental Table 1. Summary of clinical evidence in ex-vivo T cell depletion**

| **Author, year** | **Cell manipulation** | **Location** | **N** | **Age, Median (range)** | **Disease** | **Graft source** | **Conditioning regimen** | **Serotherapy** | **Post-HCT GVHD prophylaxis** | **CD34+ cells/kg** | **T subset** | **Graft failure** | **aGVHD** | **cGVHD** | **Outcome** |
| --- | --- | --- | --- | --- | --- | --- | --- | --- | --- | --- | --- | --- | --- | --- | --- |
| O, Reilly, 1987^1^ | SBA-E- | New York  (USA) | 52 | 8 (1-49) | ALL: 54% AML: 21% CML: 21% NHL: 4% | Haplo BM | TBI+CY  or TBI+Ara-C | +/- ATG | CSA: 17% PRED: 60% None: 23% | NR | NR | 30% | Gr 2-4: 6% Gr 3-4: 2% | NR | NR |
| Aversa, 1994^2^ | Megadose 1: SBA-E-BM, E-PBSC 2: SBA-E-BM&PBSC | Perugia (Italy) | 17 | Mean 23 (6-51) | ALL: 53% AML: 29% CML: 18% | Haplo BM + PBSC | TBI+TT+CY | ATG | None | 1: 13.9 x 10^6 2: 16 x 10^6 | CD3+ 1: 0.6 x 10^6 2: 0.1 x 10^6 | 6% | Gr 2-4: 6% Gr 3-4: 6% | NR | TRM: 56% Relapse: 13% |
| Aversa, 1998^3^ | SBA-E- | Perugia (Italy) | 43 | 22 (4-53) | ALL: 53% AML: 47% | Haplo BM + PBSC | TBI+TT+FLU | ATG | None | PBSC+BM: 10.6 x 10^6 PBSC only: 14.0 x 10^6 | CD3+ PBSC+BM: 0.04 x 10^6 PBSC only: 0.03 x 10^6 | 5% | 0% | 0% | TRM: 40% Relapse: 30% |
| Stern,  2008^4^ | SBA-E-  (n = 6) CD34+ retrieval  (n = 112) | Perugia, Pavia (Italy) | 118 | 1. Paternal donor:  19 (2-52) 2. Maternal donor:  17 (2-41) | ALL: 57% AML: 43% | Haplo-PBSC | TBI+TT+FLU | ATG | None | 1: 12.1 x 10^6 2: 14.5 x 10^6 | CD3+ 1: 0.01 x 10^6 2: 0.01 x 10^6 | 1: 9.9% 2: 6.4% | 1: Gr 2-4: 16.9% 2: Gr 2-4: 6.4% | 1: 16.2% 2: 12.1% | 1: 5-yr TRM: 42.3% 5-yr RM: 46.5% 2: 5-yr TRM: 26.6% 5-yr RM: 22.7% |
| Aversa, 2005^5^ | CD34+ retrieval | Perugia (Italy) | 104 | 33 (9-64) | ALL: 36% AML: 64% | Haplo-PBSC | TBI+TT+FLU | ATG | None | 13.8 x 10^6 | CD3+: 0.01 x 10^6 | 9% | Gr 2-4: 8% Gr 3-4: 2% | Limited: 3% Extended: 4% | NRM: 37% Relapse: 25% |
| Klingebiel, 2010^6^ | CD34+ retrieval | EBMT registry | 102 | 8.7 (0.6-16) | ALL | Haplo-PBSC: 96% Haplo-BM: 6% | MAC 100% - TBI 76% | ATG/ALG 75% None 22% Unknown 4% | NR | 12.3 x 10^6 | CD3+: 0.05 x 10^6 | 13% | Gr 2-4: 22% Gr 3-4: 9% | 17% | 5 yr-NRM: 37% 5 yr-relapse: 36% |
| Salzmann-Marique, 2018^7^ | CD34+ retrieval | Frankfurt (Germany) | 11 | 7.5 (3.5-23) | ALL: 82% AML: 18% | Haplo-PBSC | MAC: 82% - TBI: 55% RIC (FLU+TT+MEL): 18% | ATG: 73% OKT3: 27% | None | 15.3 x 10^6 | CD3+: 0.001 x 10^6 | NR | Gr 2-4: 45% Gr 3-4: 18% | 27% | TRM: 27% Relapse: 36% |
| Lang, 2005^8^ | CD3+/CD19+  removal | Tubingen (Germany) | 11 | Children  (unspecified) | ALL: 36% AML: 64% | Haplo-PBSC | TBI-based MAC or RIC  (MEL+FLU+TT) | OKT3 | CSA or MTX when  CD3+ >0.025 x 10^6/kg | NR | CD3+: 0.03 x 10^6 | 9% | Gr 2-4: 27% Gr 3-4: 0% | NR | TRM: 0% Relapse: 55% |
| Pérez-Martínez, 2012^9^ | CD3+/CD19+  removal | Madrid (Spain) | 15 | 9.7 +/- 1.1 (mean +/- SD) | ALL: 67% AML: 33% | Haplo-PBSC | NMA: FLU+BU+TT+mPRED | None | CSA till Day 60-100 | 9.2 x 10^6 | CD3+: 0.06 x 10^6 | 27% | Gr 1-2: 20% Gr 3-4: 13% | Limited 7% Extended 7% | NRM: 20% Relapse: 33% EFS: 46% |
| Lang, 2014^10^ | CD3+/CD19+  removal | 5 sites (Germany, Austria) | 46 | 11.1 (1.1 - 23.7) | ALL: 57% AML: 37% MDS: 7% | Haplo-PBSC | MAC: FLU+TT+MEL (CLO replaced FLU in some) | OKT3 or ATG | MMF till Day 60 when  CD3+ >0.025 x 10^6/kg | 14.5 x 10^6 | CD3+: 0.06 x 10^6 | 13% | Gr 2-4: 26% Gr 3-4: 7% | Limited 9% Extensive 12% | 5 yr TRM: 20% Relapse: 63% (median f/u 4.3 yr) |
| Diaz, 2016^11^ | CD3+/CD19+  removal | Madrid (Spain) | 75 | 9 (0.5-19) | ALL: 51% AML/MDS/JMML: 49% | Haplo-PBSC | FLU+BU+TT+mPRED | None | CSA taper <day 30 | 7.0 x 10^6 | CD3+: 0.01 x 10^6 | 13% | Gr 2-4: 23% Gr 3-4: 13% | Mild: 13% Moderate: 13% Severe: 4% | 2 yr NRM: 23% Relapse: 32% |
| Salzmann-Marique, 2018^7^ | CD3+/CD19+  removal | Frankfurt (Germany) | 29 | 10.6 (1.3-26) | ALL: 55% AML: 45% | Haplo-PBSC | MAC: 17% - TBI: 14% RIC (FLU+TT+MEL): 83% | ATG: 17% OKT3: 72% None:7% | MMF: 86% CSA+MTX: 3% None: 10% | 10.1 x 10^6 | CD3+: 0.009 x 10^6 | NR | Gr 2-4: 21% Gr 3-4: 14% | 7% | TRM: 17% Relapse: 31% |
| Lang,  2015^12^ | TCRαβ+/CD19+ removal | Tubingen (Germany) Graz (Austria) | 41 | 9 (2-18) | ALL: 49% AML: 7% MDS/JMML: 10% Solid tumor: 12% | Haplo-PBSC | FLU+TT+MEL or CLO+TT+MEL | OKT3 or ATG | MMF (till Day 30) | 14.9 x 10^6 | ab+: 0.02 x 10^6 gd+: 11.0 x 10^6 | 12% | Gr 2-4: 24% Gr 3-4: 15% | Limited: 15% Extensive: 7% | OS 51% |
| Locatelli,  2017^13^ | TCRαβ+/CD19+ removal | Rome  (Italy) | 80 | 9.7 (0.9-20.9) | ALL: 70% AML: 30% | Haplo-PBSC | TBI+TT+FLU: 50% TBI+TT+MEL: 25% TT+BU+FLU: 16% BU+CY+MEL: 9% | ATLG Rituximab | None | 13.9 x 10^6 | ab+: 0.05 x 10^6 gd+: 8.1 x 10^6 | 3% | Gr 1-2 (skin): 30% Gr 3-4: 0% | Limited (skin) 5% | 5yr-NRM: 5% Relapse: 24% 5yr-GRFS: 71% |
| Bertaina,  2018^14^ | TCRαβ+/CD19+ removal | 6 sites  (Italy) | 98 | 6.6 (0.1-17.3) | ALL: 68% AML: 32% | Haplo-PBSC | TBI-based MAC: 74% BU-based MAC: 18% TREO-based MAC: 7% Other MAC: 1% | ATLG Rituximab | None | 14.4 x 10^6 | ab+: 0.04 x 10^6 gd+: 8.1 x 10^6 | 2% | Gr 2 (skin): 16% Gr 3-4: 0% | Limited: 5% Extensive: 1% | NRM: 9% Relapse: 29% 5-yr GRFS: 58% |
| Erby, 2018^15^ | TCRαβ+/CD19+ removal | Istanbul (Turkey) | 21 | Mean 10.9 | ALL: 67% AML: 33% | Haplo-PBSC | FLU+TT+MEL | ATG Rituximab | MMF +/- CSA | NR | NR | 14% | Gr 2-4: 33% | 19% | TRM: 14% Relapse: 10% |
| Shelikhova, 2019^16^ | TCRαβ+/CD19+ removal | Moscow  (Russia) | 22 | 9.6 (1-18) | AML (100%) | Haplo-PBSC | TREO+MEL  or TREO+TT | Rituximab, bortezomib,  tocilizumab or abatacept | None | 8.5 x 10^6 | ab+: 0.01 x 10^6 gd+: 8.0 x 10^6 | 9% | Gr 2-4: 18% Gr 3-4: 14% | Moderate: 14% Severe: 9% | 2 yr-TRM: 9% 2 yr-Relapse: 42% |
| Maschan, 2020^17^ | TCRαβ+/CD19+ removal | Moscow  (Russia) | 37 | 5.6 (0.4-23) | AML (100%) | Haplo-PBSC | TREO+FLU+MEL  or TREO+FLU+TT | ATG | Tacrolimus (till Day 30)+MTX or Bortezomib | 9 x 10^6 | ab+: 0.01 x 10^6 gd+: 11 x 10^6 | 0% | [Haplo + MUD] Gr2-4: 19% Gr 4: 0% | [Haplo + MUD] Total: 22% Mod/sev: 7% | 3 yr-TRM 5% 3 yr-relapse 14% 3 yr-GRFS 70% |
| Pérez-Martínez, 2020^18^ | TCRαβ+/CD19+ removal | 10 sites (Spain) | 34 | [All TCD]  9.1 (IQR: +/-7.9) | [All TCD]  ALL: 54% AML: 34% MDS: 6% Others: 6% | Haplo-PBSC | 1: FLU+MEL+TT 2: BU+FLU +/- TT | ATG | MMF | [All TCD]  6.8 x 10^6 | NR | 6% | Gr 1-2: 27% Gr 3-4: 15% | 2 yr total: 16% | TRM: 15% 2 yr Relapse: 28% 2 yr GRFS: 53% |
| Triplett, 2018^19^ | CD34+ retrieval CD45RA+ removal NK cell infusion | Memphis (USA) | 26 | 10.5 (0.6-20.7) | ALL: 42% AML: 42% MDS: 4% Others: 12% | Haplo-PBSC | FLU+TT+MEL+TLI | None | MMF till Day<60 | 15.6 x 10^6 | CD3+: 80.0 x 10^6 CD3+CD45RA+: 0.011 x 10^6 | NR | Gr 3-4: 23% | NR | 1.5 yr TRM 15% |

Note: NR (not reported) indicate the information was unable in the article.
Abbreviations: ALG = antilymphocyte globulin; ALL = acute lymphoblastic leukemia; AML = acute myeloid leukemia; Ara-C = cytarabine; ATG = antithymocyte globulin; ATLG = anti-T lymphocyte globulin; BM =bone marrow; BU = busulfan; CLO = clofarabine; CML = chronic myeloid leukemia; CR = complete remission; CSA = cyclosporine A; CY = cyclophosphamide; FLU = fludarabine; GVHD = graft-versus-host disease [a = acut, c = chroic]; Gr = grade; GRFS = GVHD-free, relapse-free survival; haplo = haploidentical, HCT = hematopoietic cell transplatation; IQR = iterquartile range; JMML = juvenile myelomonocytic leukemia; MAC = myeloablative conditioning; MDS = myelodysplastic syndrome; MEL = melphalan; MMF = mycophenolate; MTX = methotrexate; MUD = matched unrelated donor; NHL = non-Hodgkin lymphoma; NK = natural killer; NMA = nonmyeloablative conditioning; NRM = nonrelapse mortality; PBSC = peripheral blood stem cell; mPRED = methylprednisolone; RAEB = refractory anemia with excess blasts; RIC = reduced intensity conditioning; RM = relapse mortality; SBA-E- = soybean agglutination negative E-rosetting negative; SD = standard deviation; TBI = total body irradiation; TCD =T cell depletion; TLI = total lymphoid irradiation; TREO = treosulfan; TRM = treatment related mortality; TT = thiotepa; USA = United States of America

**References:**

1. O'Reilly RJ, Keever C, Kernan NA, et al. HLA nonidentical T cell depleted marrow transplants: a comparison of results in patients treated for leukemia and severe combined immunodeficiency disease. *Transplant Proc* 1987;19(6 Suppl 7):55-60.

2. Aversa F, Tabilio A, Terenzi A, et al. Successful engraftment of T-cell-depleted haploidentical "three-loci" incompatible transplants in leukemia patients by addition of recombinant human granulocyte colony-stimulating factor-mobilized peripheral blood progenitor cells to bone marrow inoculum. *Blood* 1994;84(11):3948-55.

3. Aversa F, Tabilio A, Velardi A, et al. Treatment of High-Risk Acute Leukemia with T-Cell–Depleted Stem Cells from Related Donors with One Fully Mismatched HLA Haplotype. *New England Journal of Medicine* 1998;339(17):1186-93. doi: 10.1056/nejm199810223391702

4. Stern M, Ruggeri L, Mancusi A, et al. Survival after T cell-depleted haploidentical stem cell transplantation is improved using the mother as donor. *Blood* 2008;112(7):2990-5. doi: 10.1182/blood-2008-01-135285 [published Online First: 20080520]

5. Aversa F, Terenzi A, Tabilio A, et al. Full haplotype-mismatched hematopoietic stem-cell transplantation: a phase II study in patients with acute leukemia at high risk of relapse. *J Clin Oncol* 2005;23(15):3447-54. doi: 10.1200/jco.2005.09.117 [published Online First: 20050307]

6. Klingebiel T, Cornish J, Labopin M, et al. Results and factors influencing outcome after fully haploidentical hematopoietic stem cell transplantation in children with very high-risk acute lymphoblastic leukemia: impact of center size: an analysis on behalf of the Acute Leukemia and Pediatric Disease Working Parties of the European Blood and Marrow Transplant group. *Blood* 2010;115(17):3437-46. doi: 10.1182/blood-2009-03-207001 [published Online First: 20091229]

7. Salzmann-Manrique E, Bremm M, Huenecke S, et al. Joint Modeling of Immune Reconstitution Post Haploidentical Stem Cell Transplantation in Pediatric Patients With Acute Leukemia Comparing CD34(+)-Selected to CD3/CD19-Depleted Grafts in a Retrospective Multicenter Study. *Front Immunol* 2018;9:1841. doi: 10.3389/fimmu.2018.01841 [published Online First: 20180814]

8. Lang P, Schumm M, Greil J, et al. A comparison between three graft manipulation methods for haploidentical stem cell transplantation in pediatric patients: preliminary results of a pilot study. *Klin Padiatr* 2005;217(6):334-8. doi: 10.1055/s-2005-872529

9. Pérez-Martínez A, González-Vicent M, Valentín J, et al. Early evaluation of immune reconstitution following allogeneic CD3/CD19-depleted grafts from alternative donors in childhood acute leukemia. *Bone Marrow Transplant* 2012;47(11):1419-27. doi: 10.1038/bmt.2012.43 [published Online First: 20120312]

10. Lang P, Teltschik HM, Feuchtinger T, et al. Transplantation of CD3/CD19 depleted allografts from haploidentical family donors in paediatric leukaemia. *Br J Haematol* 2014;165(5):688-98. doi: 10.1111/bjh.12810 [published Online First: 20140304]

11. Diaz MA, Pérez-Martínez A, Herrero B, et al. Prognostic factors and outcomes for pediatric patients receiving an haploidentical relative allogeneic transplant using CD3/CD19-depleted grafts. *Bone Marrow Transplant* 2016;51(9):1211-6. doi: 10.1038/bmt.2016.101 [published Online First: 20160418]

12. Lang P, Feuchtinger T, Teltschik HM, et al. Improved immune recovery after transplantation of TCRαβ/CD19-depleted allografts from haploidentical donors in pediatric patients. *Bone Marrow Transplantation* 2015;50(2):S6-S10. doi: 10.1038/bmt.2015.87

13. Locatelli F, Merli P, Pagliara D, et al. Outcome of children with acute leukemia given HLA-haploidentical HSCT after αβ T-cell and B-cell depletion. *Blood* 2017;130(5):677-85. doi: 10.1182/blood-2017-04-779769 [published Online First: 20170606]

14. Bertaina A, Zecca M, Buldini B, et al. Unrelated donor vs HLA-haploidentical α/β T-cell– and B-cell–depleted HSCT in children with acute leukemia. *Blood* 2018;132(24):2594-607. doi: 10.1182/blood-2018-07-861575

15. Erbey F, Akçay A, Atay D, et al. Comparison of outcomes after HLA-matched unrelated and αβ T-cell-depleted haploidentical hematopoietic stem cell transplantation for children with high-risk acute leukemia. *Pediatric Transplantation* 2018;22(4):e13192. doi: <https://doi.org/10.1111/petr.13192>

16. Shelikhova L, Ilushina M, Shekhovtsova Z, et al. αβ T Cell-Depleted Haploidentical Hematopoietic Stem Cell Transplantation without Antithymocyte Globulin in Children with Chemorefractory Acute Myelogenous Leukemia. *Biology of Blood and Marrow Transplantation* 2019;25(5):e179-e82. doi: <https://doi.org/10.1016/j.bbmt.2019.01.023>

17. Maschan M, Shelikhova L, Ilushina M, et al. Outcome of αβ T cell-depleted transplantation in children with high-risk acute myeloid leukemia, grafted in remission. *Bone Marrow Transplantation* 2020;55(1):256-59. doi: 10.1038/s41409-019-0531-3

18. Pérez-Martínez A, Ferreras C, Pascual A, et al. Haploidentical transplantation in high-risk pediatric leukemia: A retrospective comparative analysis on behalf of the Spanish working Group for bone marrow transplantation in children (GETMON) and the Spanish Grupo for hematopoietic transplantation (GETH). *American Journal of Hematology* 2020;95(1):28-37. doi: <https://doi.org/10.1002/ajh.25661>

19. Triplett BM, Muller B, Kang G, et al. Selective T-cell depletion targeting CD45RA reduces viremia and enhances early T-cell recovery compared with CD3-targeted T-cell depletion. *Transpl Infect Dis* 2018;20(1) doi: 10.1111/tid.12823 [published Online First: 20180116]
